# Malignancy during pregnancy in Japan: an exceptional opportunity for early diagnosis

**DOI:** 10.1186/s12884-018-1678-4

**Published:** 2018-02-08

**Authors:** Masayuki Sekine, Yoshiyuki Kobayashi, Tsutomu Tabata, Tamotsu Sudo, Ryuichiro Nishimura, Koji Matsuo, Brendan H. Grubbs, Takayuki Enomoto, Tomoaki Ikeda

**Affiliations:** 1 Department of Obstetrics and Gynecology, Niigata University Graduate School of Medical and Dental Science, 1-757 Asahimachi-dori, Niigata, 951-8510 Japan; 2 Departments of Obstetrics and Gynecology, Mie University Graduate School of Medicine, Mie, Japan; 3 Department of Gynecology, Hyogo Cancer Center, Hyogo, Japan; 4 Department of Obstetrics and Gynecology, University of Southern California, Los Angeles, CA USA

**Keywords:** Malignancy, Pregnancy, Cervical cancer, Early diagnosis

## Abstract

**Background:**

Malignancy during pregnancy has become a significant cause of maternal death in developed countries, likely due to both an older pregnant population, and increases of cervical cancer in younger women. Our aim is to investigate the clinical aspects of malignancy during pregnancy in Japan and to use this information to identify opportunities for earlier detection and treatment.

**Methods:**

We provided a questionnaire to 1508 secondary or tertiary care hospitals in Japan. We reviewed the clinical characteristics of cases with malignancy during pregnancy for the period of January to December, 2008. From the 760 institutions which responded, we obtained clinical information for 227 unique cases. The questionnaire provided clinical information, including disease site, pregnancy outcome and how the disease was detected.

**Results:**

The most common type of malignancy was cervical cancer (*n* = 162, 71.4%) followed by ovarian (*n* = 16, 7.0%) and breast cancer (*n* = 15, 6.6%). Leukemia (*n* = 7, 3.1%), colon cancer (*n* = 5, 2.2%), gastric cancer (*n* = 5, 2.2%), malignant lymphoma (*n* = 4, 1.8%), thyroid cancer (*n* = 3, 1.3%), brain cancer (*n* = 3, 1.3%), endometrial cancer (*n* = 2, 0.9%), and head and neck cancer (*n* = 2, 0.9%) accounted for the remaining cases. Overall, gynecological malignancies accounted for 79.3% (95% confidence interval 74.0–84.6) of pregnancy associated malignancies diagnosed in the present study. The majority of cervical cancers, 149 (92.0%) of 162, were diagnosed by a Pap (Papanicolaou) smear during early gestation. Ten (62.5%) of the ovarian cancer cases were diagnosed by ultrasonography during a prenatal checkup or at the time of initial pregnancy diagnosis. Out of 14 breast cancers, only one (7.1%) was diagnosed by screening breast exam.

**Conclusions:**

From this study, we reaffirm the clear and significant benefits of prenatal checkups starting at an early gestational age for the detection of gynecological cancers during pregnancy. Conversely, breast cancer detection during pregnancy was poor, suggesting new strategies for early identification of this disease are required.

## Background

Malignancy during pregnancy has recently become a major cause of maternal death in developed countries. The incidence of malignancies coinciding with pregnancy increased from 1:2000 in 1964 to 1:1000 deliveries in 2000 [1–4]. The increase is attributed to not only higher rates of cancer in general but also to delays in childbearing to the third and fourth decades of life for women [5]. This is also associated with increase in the incidence rate of cervical cancer in 20 to 49-year-olds has been seen in Japan [6–8]. This is assumed to be a result of a decline in the age of initial incidence of HPV (Human papillomavirus) infection due to a decline in the age of first sexual intercourse, in addition to low screening rate.

It is noteworthy that a nationwide investigation of pregnancy-linked malignancy has yet to be performed in Japan, so the underlying causes of this increase are uncertain. Several reports on malignancies during pregnancy have been published [3, 9–13] (e.g. Cancer Statistics of American Cancer Society’s Epidemiology Research Program, a population-based cohort study from the Cancer Registry and the Medical Birth Registry of Norway, and an international collaborative setting of institutional registry in Belgium, the Netherlands and Czech Republic). In these reports, gynecological tumors are among the malignancies most frequently diagnosed during pregnancy [9–12], particularly those of cervical and ovarian origin [13]. When managing such tumors, the physician must consider both potential fetal effects, as well as the potential loss of the patient’s future reproductive capacity as a result of any chosen cancer therapy.

In this study, we have investigated the clinical characteristics of malignancy during pregnancy in Japan, with the goal that our findings will contribute to the earlier detection and better management of malignant diseases during pregnancy.

## Methods

This study was performed under ethics committee approval of National Cerebral and Cardiovascular Center in Japan. We developed a questionnaire to investigate the clinical characteristics of all cases of pregnancy associated malignancy and distributed copies of this questionnaire to all training hospitals within the Japanese Society of Obstetrics and Gynecology (1475 institutions) and the Japanese Association of Clinical Cancer Centers (32 institutions). Most of the cases has been collected in hospital-based tumor registries. Subsequently, the attending obstetrician or gynecologist has examined the clinical information of the cases from medical records. Over the period of January to December 2008, 760 responding institutions provided information for 227 relevant cases, which we analyzed for clinical characteristics including the site of disease, method of disease detection, and pregnancy outcome.

## Results

The clinical backgrounds and obstetrical characteristics of the 227 malignant cases reported during pregnancy for this study are shown in Table 1. The median age of the cases was 31.0 years (range: 14–41); 94 patients (41.4%) were nulliparous, 130 (57.3%) were primiparous or multiparous, and 3 were unknown. Pregnancy outcomes were available for all 227 of the cases, and 133 (58.6%) of them delivered at term. As shown in Table 1, the remaining pregnancies resulted in either: iatrogenic preterm delivery (18.9%), elective termination (10.6%), spontaneous abortion (5.3%), or spontaneous preterm delivery (4.4%).

**Table 1:** Obstetrical characteristics of malignancy during pregnancy (*n* = 227)

age (range)	31.0	(14–41)
parity		
nulliparaous	94	(41.4%)
multiparaous	130	(57.3%)
unknown	3	(1.3%)
pregnancy outcome		
abortion		
artificial	24	(10.6%)
spontanious	12	(5.3%)
preterm delivery		
iatrogenic	43	(18.9%)
spontanious	10	(4.4%)
term delivery	133	(58.6%)
unknown	5	(2.2%)

**Fig. 1:**
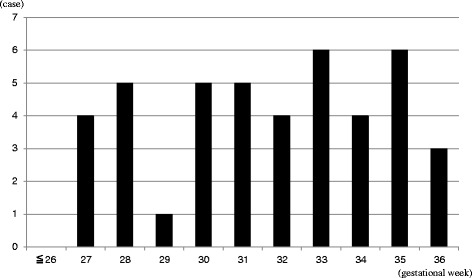
Distribution of gestational age at induced termination after 22 weeks gestation (*n* = 43). There was no case of an induced termination before 27 weeks gestation. The gestational age of termination was almost equally divided from 27 weeks until 36 weeks gestation

The distribution of gestational age at iatrogenic preterm delivery (after 22 weeks of gestation) is shown in Fig. 1. None occured between 22 and 27 weeks gestation. The gestational age at delivery was distributed almost uniformally from 27 weeks until 36 weeks.

**Fig. 2:**
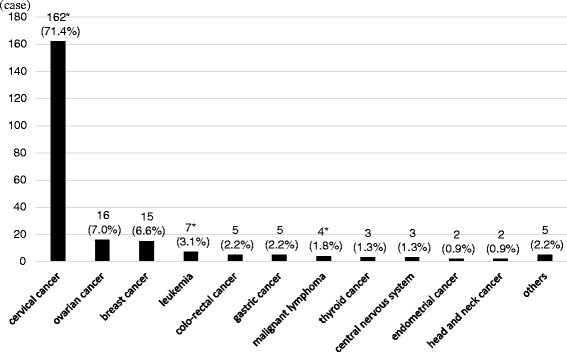
Site of malignant disease during pregnancy (*n* = 227). Most cases were cervical cancer (162 out of 227, 71.4%), ovarian cancer (16 cases, 7.0%), and breast cancer (15 cases, 6.6%). *Two cases with cervical cancer were affected with other malignancy; leukemia or malignant lymphoma, respectively

The majority of cases identified were cervical cancer (71.4%) followed by ovarian cancer (7.0%) and breast cancer (6.6%). Small numbers of malignancies at various sites account for the remaining cases as seen in Fig. 2. Overall, gynecological malignancies accounted for 79.3% (95% confidence interval 74.0–84.6) of pregnancy associated cancer diagnosed in the present study. The stage at diagnosis of 162 cases with cervical cancer in this study was as follows: 102 cases (63%) in CIN3 (cervical intraepithelial neoplasia: CIN), 16 cases (10%) in stage Ia, 33 cases (20%) in stage Ib, 5 cases (3%) in stage II, 2 cases (1%) in stage IV and 4 cases (3%) with unknown clinical stage. The histologic type of 16 cases with ovarian cancer in this study was as follows: 5 cases with adenocarcinoma (2 endometrioid, 1 serous, 1 clearcell, and 1 mucinous type), 2 with serous borderline tumor, 4 with germcell tumor (3 immature teratoma and 1 dysgerminoma), 1 with malignant transformation of mature teratoma, 1 with sertoli-leidich tumor, and 3 with unknown histology.

Table 2 demonstrates how the most common types of malignancy identified during pregnancy were diagnosed. Routine Pap (Papanicolaou) smear screening detected 92.0% of the cervical cancer cases, with the remainder identified due to vaginal bleeding, abnormal discharge, or abdomino-pelvic pain. Over half of the ovarian cancer cases (62.5%) were incidentally diagnosed by ultrasonography performed as part of a routine fetal assessment. Three cases (18.8%) were diagnosed at the time of a Caesarean section, with the remaining 3 cases identified either due to abdominal distention or palpation of swollen lymph nodes. Only one of the breast cancer cases (7.1%) was identified by a healthcare provider at the time of routine screening. The remainder were identified by patients performing self-examinations.

**Table 2:** Opportunity to detect malignancy during pregnancy

Malignancy during pregnancy	number of cases (%)
Cervical cancer (*n* = 162)	
screening Pap cytology	149 (92.0%)
abnormal vaginal bleeding	11 (6.8%)
abnormal vaginal discharge	1 (0.6%)
abdomino-pelvic pain	1 (0.6%)
Ovarian cancer (*n* = 16)	
ultrasonography (routine prenatal care)	10 (62.5%)
incidental (during cesarian section)	3 (18.8%)
abdominal distension	2 (12.5%)
abnormal lymphadenopathy	1 (6.3%)
Breast cancer (*n* = 14)	
self-detection of a palpable mass	13 (92.9%)
health care screening	1 (7.1%)

## Discussion

In our study, we found that gynecological malignancies accounted for approximately 80% of all malignant diseases with pregnancy during 2008. The most common pregnancy associated malignancies worldwide are cervical cancer, breast cancer, lymphoma, ovarian cancer, and melanoma [12, 14, 15]. Of these, cervical and breast cancers account for 50% of all cancers occurring during pregnancy [15], which is a lower rate than is seen in the present study (78%). The obstetrician will often have the best opportunity to make the diagnosis of malignancy during pregnancy, so awareness of the associated symptoms is required during regular pre-natal checkups.

We found that conducting a Pap smear during the early pregnancy period was very effective in early detection of cervical cancer, the most common pregnancy associated cancer in Japan. The prevalence of cervical cancer for women in their twenties and thirties has risen dramatically over the past decade in several studies in Japan [6–8]. Based on the findings of this study, it is essential that the obstetrician ask each pregnant patient about her past Pap smear and examination history and strongly recommend this test for any patient who is not up to date on her screening.

In order to detect ovarian cancer during pregnancy, assessment of the adnexae is important at the time of all prenatal ultrasounds. In review of the literature, up to one third of ovarian cancers diagnosed during pregnancy were identified incidentally by ultrasonography, making it the most common method of tumor detiction [16–18]. As gestational age increases, use of transabdominal ultrasound observation to detect an ovarian tumor becomes more difficult, so this is particularly important at the time of the first trimester examination. In cases where the ovaries are not adequately visualized, or characterized by transabdominal ultrasound, a vaginal probe can often provide a better assessment.

In some cases MRI (Magnetic Resonance Imaging) subsequent to an unclear or suspicious ultrasound finding may be necessary to help differentiate whether an ovarian mass is malignant or benign [19]. As the progression of ovarian cancer can be very rapid, at our institution we use a combination of early pregnancy vaginal ultrasonography with a follow-up MRI scan in all cases where there is any suspicion of malignancy.

We found that the majority of breast cancer cases were found following self-detection of a palpable mass and not by a health care provider. Increased breast cancer awareness during pregnancy may contribute to this finding. Additionally, pregnant women are generally not yet at an age where routine mammography is recommended, skewing the results towards initial breast tumor discovery by self-examination. Regular prenatal checkups did not appear to be useful for detecting breast masses, however this may be an area where improvement may come from stressing the importance of a thorough examination, with appropriate close follow up of any suspicious findings. It is a general practice in Japan for midwives, rather than obstetricians to perform breast examinations during prenatal care. Thus, several strategies may be needed to improve the early detection of breast cancer during pregnancy. These might include development of a universal training guideline for breast examination by obstetricians with possible assistance by the midwife service.

## Conclusions

From this study, we reaffirm the significant benefits of pre-natal checkups at an early gestational age for early detection of gynecologic cancer during pregnancy. On the other hand, the detection of non-gynecologic cancers tends to be delayed, and it is clear that we need new strategies as to how to improve screening, particularly for breast cancer in pregnant women.

## Abbreviations

CIN: Cervical intraepithelial neoplasia
HPV: human papillomavirus
MRI: Magnetic resonance imaging
Pap: Papanicolaou
